# Evaluation of Chimpanzee Adenovirus and MVA Expressing TRAP and CSP from *Plasmodium cynomolgi* to Prevent Malaria Relapse in Nonhuman Primates

**DOI:** 10.3390/vaccines8030363

**Published:** 2020-07-06

**Authors:** Young Chan Kim, Barbara Dema, Roberto Rodriguez-Garcia, César López-Camacho, Fabiana M. S. Leoratti, Amar Lall, Edmond J. Remarque, Clemens H. M. Kocken, Arturo Reyes-Sandoval

**Affiliations:** 1 The Jenner Institute, University of Oxford, Old Road Campus Research Building, Roosevelt Drive, Oxford OX3 7DQ, UK; young.kim@some.ox.ac.uk (Y.C.K.); barbara.demajimenez@ndm.ox.ac.uk (B.D.); cesarlc@well.ox.ac.uk (C.L.-C.); fabiana.leoratti@gmail.com (F.M.S.L.); amar.s.lall@gmail.com (A.L.); 2 Department of Parasitology, Biomedical Primate Research Centre (BPRC), 2288 GJ Rijswijk, The Netherlands; rodriguez@bprc.nl (R.R.-G.); remarque@bprc.nl (E.J.R.); kocken@bprc.nl (C.H.M.K.)

**Keywords:** *P. vivax*, Circumsporozoite protein, CSP, thrombospondin related adhesive protein: TRAP, adenoviruses, MVA, malaria, vaccines

## Abstract

*Plasmodium vivax* is the world’s most widely distributed human malaria parasite, with over 2.8 billion people at risk in Asia, the Americas, and Africa. The 80–90% new *P. vivax* malaria infections are due to relapses which suggest that a vaccine with high efficacy against relapses by prevention of hypnozoite formation could lead to a significant reduction in the prevalence of *P. vivax* infections. Here, we describe the development of new recombinant ChAdOx1 and MVA vectors expressing *P. cynomolgi* Thrombospondin Related Adhesive Protein (PcTRAP) and the circumsporozoite protein (PcCSP). Both were shown to be immunogenic in mice prior to their assessment in rhesus macaques. We confirmed good vaccine-induced humoral and cellular responses after prime-boost vaccination in rhesus macaques prior to sporozoite challenge. Results indicate that there were no significant differences between mock-control and vaccinated animals after challenge, in terms of protective efficacy measured as the time taken to 1st patency, or as number of relapses. This suggests that under the conditions tested, the vaccination with PcTRAP and PcCSP using ChAdOx1 or MVA vaccine platforms do not protect against pre-erythrocytic malaria or relapses despite good immunogenicity induced by the viral-vectored vaccines.

## 1. Introduction

*Plasmodium vivax* is considered to be the biggest hurdle towards malaria eradication. This protozoa is the most widespread malaria parasite in humans, as well as the most difficult to eliminate from endemic countries in Asia, the Americas and Africa. It accounts for 132–391 million clinical infections of *P. vivax* each year and over 2.8 billon people are considered to be at risk of *P. vivax* transmission [1,2]. *P. vivax* as well as *P. cynomolgi*, have a sophisticated ability to hide in the infected host for long periods of time by developing structures in hepatocytes known as hypnozoites [3]. These dormant forms change dramatically the epidemiological landscape of malaria, as reactivation by unknown mechanisms within days, months or years after the first contact with the parasite, causing a relapse and the onset of malaria symptoms in complete absence of mosquitoes [3,4]. It has been shown that relapses are responsible for 80–90% new *P. vivax* malaria infections [5,6], which suggests that targeting hypnozoites either by preventing its formation or promoting elimination from the liver may lead to significant reductions in *P. vivax* transmission [7]. Ivo Mueller et al. have also suggested that a vaccine with high efficacy against relapses by prevention of hypnozoite formation would be major achievement as it would result in significant reduction in *P. vivax* prevalence and infections, leading to a decrease in transmission [7]. 

Despite many years of research, the only drug licensed for the radical cure and relapse prevention of *P. vivax*, primaquine, can trigger severe hemolytic anemia in glucose-6-phosphate dehydrogenase (G6PD) deficient individuals, making it necessary to test a patient for this common genetic disorder that is quite common in endemic areas, before prescribing the drug [8]. Novel compounds targeting the hypnozoite are urgently needed to replace primaquine [9]. We have previously demonstrated that adenoviral vectors expressing the malaria circumsporozoite peptide (Pb9) induce high CD8+ T cell frequencies that can be successfully deployed to the liver to eliminate the sporozoite-infected hepatocytes, raising the possibility that this vaccination approach could stand a good chance to eliminate the hypnozoite forms [10]. Recent data by the group of Stefan Kappe, indicate that membranes surrounding hypnozoites contain the circumsporozoite protein, raising the possibility of becoming a target by cytotoxic lymphocytes (CTLs) [11]. TRAP (thrombospondin-related anonymous protein) mediates the invasion of sporozoites into the hepatocytes. Viral vectored vaccines targeting TRAP have shown CD8+ and antibody-mediated-protection against sporozoites of *P. berghei* expressing *P. vivax* proteins [12]. Similarly, we have previously described that co-administration of adenovirus and MVA expressing circumsporozoite protein (CSP) and TRAP conferred high levels of protection to mice challenged with *P. berghei* sporozoites [13]. The aim of this study was to determine whether the immunization of Nonhuman Primates (NHP) with two vaccine candidates based on the *P. cynomolgi* Thrombospondin Related Adhesive Protein (PcTRAP) and the circumsporozoite protein (PcCSP), can prevent relapse through the induction of immune responses to prevent formation or to target hypnozoite-bearing hepatocytes. We vaccinated rhesus macaques with an adenovirus from chimpanzee origin, ChAdOx1 and Modified Vaccinia Ankara, or MVA expressing PcTRAP and PcCSP in a prime-boost regimen and assessed vaccine-elicited immunogenicity and efficacy. We confirmed good vaccine-induced humoral and cellular responses after prime-boost vaccination of rhesus macaques prior to sporozoite challenge. However, there were no significant differences between mock-control and vaccinated animals after challenge, in terms of protective efficacy measured as the time taken to 1st patency, or as number of relapses. This suggests that under the conditions tested, the vaccination with PcTRAP and PcCSP using ChAdOx1 or MVA vaccine platforms do not protect against pre-erythrocytic malaria or relapses in a challenge with 10,000 *P. cynomolgi* sporozoites.

## 2. Materials and Methods

### 2.1. Antigen Design in ChAdOx1 and MVA Vaccines Expressing TRAP and the CSP from Plasmodium cynomolgi

Two genes from *Plasmodium cynomolgi*, the Circumsporozoite Protein (CSP) and Thrombospondin Related Adhesive Protein (TRAP) were synthesized by Life Technologies using the annotated sequences of CSP from *P. cynomolgi* strain B (Uniprot P08676; GeneBank AAA29539.1) and TRAP (Uniprot 044019; GeneBank CAA73140.1). Endogenous leading sequences from both genes were replaced by the tPA (tissue plasminogen activator signal peptide) sequence to improve expression and a Kozac sequence to enhance translation. The tPA facilitates the protein transport from the endoplasmic reticulum (RE) to the Golgi apparatus, thus increasing the expression and secretion of the antigen [14,15,16]. The transgenes were ligated to pENTR4-Mono backbone plasmid after KpnI/NotI digestion and expanded in *Escherichia coli.* Upon cloning into ChAdOx1 to form ChAdOx1 PcCSP, the viral vector was produced in HEK293T cells and purified by Cesium Chloride to reach a concentration of 8.9 × 10^9^ infectious units (IU)/mL and 8.1 × 10^11^ viral particles (vp)/mL corresponding to a particle to infectious units (P:I) ratio of 91. ChAdOx1 PcTRAP was produced and purified under similar conditions and obtained at a concentration of 3.1 × 10^10^ IU/mL and 2.3 × 10^12^ vp/mL, corresponding to a P:I of 73. The sterility of the virus was also confirmed by inoculation of TSB broth with 10 L of purified virus and incubation for 3 days at 35 °C. To generate the MVA constructs, the transgenes were ligated to the MVA backbone after KpnI/XhoI restriction enzyme digestion. MVA PcCSP was produced in primary cell cultures of chicken embryo fibroblasts (CEF) at a final yield of 2.1 × 10^9^ plaque-forming units (PFU)/mL, and MVA PcTRAP was produced under similar conditions and purified at a concentration of 1.3 × 10^9^ PFU/mL. Viral vectors were stored in 10 mM Tris, 140 mM NaCl, pH 7.8 as formulation buffer.

### 2.2. Design, Production and Purification of PvCSP and PvTRAP Proteins in HEK293T Cells 

The PcCSP was amplified by PCR with the following primers: ACCGGTCACAACGTGGACTTCTCCA (Forward) and GGTACCCTTGTCCATGGTGCACACTTC (Reverse). Sequence underlined correspond to the addition of restriction sites (AgeI and KpnI) that allowed the cloning into the PhLSec expression vector. Amplification started from position 24 a.a. (lacking the endogenous signal sequence) to position 340 a.a. (lacking the endogenous transmembrane domain). The PcTRAP sequence was amplified by PCR with the following primers: ACCGGTGGCGACCAGAAAATCGTGGA (Forward) and GGTACCGGAGTTGTTTGGGATGTCGC (Reverse). Sequence underlined correspond to the addition of restriction sites (AgeI and KpnI) that allowed the cloning into the PhLSec expression vector. Amplification started from position 25 a.a. (lacking the endogenous signal sequence) to position 496 a.a. (lacking the endogenous C-terminal domain). Resulting PCR-cloning products were ligated with AgeI and KpnI into the PhLSec plasmid that has a chicken b-actin/rabbit b-globin hybrid promoter with a signal secretion sequence and a Lys-His6 tag [17]. The recombinant DNA plasmids were purified from *E. coli* using the miniprep kit (Qiagen, Hilden, Germany) and each of plasmid was verified by restriction analysis and DNA sequencing (Source, BioScience). The resultant expression PhHLSec plasmid was amplified using QIAGEN Giga Preps (Qiagen, Germany). The mammalian expression of PcCSP and PcTRAP were carried out in roller bottles using the standard PEI transfection of HEK293T cells according to the protocol published previously [18]. Briefly, the PhHLSec PcCSP or PcTRAP plasmid (500 µg) was transfected in HEK-293T cells using polyethyleneimine (PEI) in roller bottles under standard cell culture conditions. Five days after transfection, cells were discarded, and media was filtered through 0.22 µM disposable filters. The secreted protein was purified from the supernatant by Ni Sepharose affinity chromatography (HisTRAPTM, GE Healthcare), using the Äkta Start chromatography system and eluted with imidazole 500 mM. Finally, the eluted protein was dialyzed using Slide-A-LyzerTM cassette (Thermo Fisher Scientific, Rockford, IL. USA) against 1×PBS.

### 2.3. Ethics Statement and Immunization in Mice 

Female C57BL/6, 6-8 weeks old mice were purchased from Envigo and used for potency assay (*n* = 3 mice per group). All animals used for this study were in accordance with UK Home Office Animals Act Project license. Procedures were approved by the University of Oxford Animal care and Ethical Review Committee (PPL 30/2414). The experimental design considered the 3Rs (replacement, reduction and refinement). Groups of mice (*n* = 3) were injected intramuscularly with either ChAdOx1 PcCSP or ChAdOx1 PcTRAP at a concentration of 1 × 10^8^ infectious units (IU). Similarly, MVA PcCSP and MVA PcTRAP were evaluated for immunogenicity in the same animal model, at concentrations of 1 × 10^6^ plaque-forming units (PFU) per mouse. ChAdOx1/MVA prime-boost (8 weeks apart) was also assessed in C57Bl/6 using the same concentration as described above. A blood sample was withdrawn at week 2 after prime or prime/boost. 

### 2.4. Ex-Vivo IFNγ ELISpot Assay

ELISpot was performed according to the methods previously published [18,19]. Briefly, MAIP ELISpot plates (Millipore UK, LTD) were coated with anti-mouse IFNγ antibody (mAb AN18, Mabtech), after 1h blocking with complete DMEM media (10% FCS). Erythrocytes were lysed using an ACK lysing buffer and leucocytes were stimulated with peptide pools spanning the whole sequence of PcCSP or PcTRAP. Twenty-mer specific PcCSP and PcTRAP structural peptides overlapped by 10 a.a. (10 μg/mL) and 2.5 × 10^5^ splenocytes from naïve mice per well. After 16 h of incubation, cells were discarded, and plates washed with PBS. Following this, 50 μL of biotinylated anti-mouse IFNγ mAb (mAb R4-6A2, Mabtech) (1:1000 in PBS) was added to each well and incubated for 2 h. After washing, plates were incubated with 50 μL of Streptavidin-ALP (Mabtech) at 1:1000 dilution in PBS for 1 h. After another washing step, developing solution (BioRad, Watford H., U.K.) was used. Once spots were visible, the reaction was stopped by rinsing the plates with water. Spots were acquired using an ELISpot reader. Spot Forming Cells (SFC)/10^6^ PBMCs producing IFNγ were calculated. For macaque samples, monkey IFN-γ ELISpot kit (3421M-2A, MABTECH) was used to determine Spot Forming Cells (SFC)/10^6^ PBMCs producing IFNγ according to the protocol. The ELISpot values were analyzed by determination of *p*-values by Kruskal–Wallis test and Dunn’s multiple comparisons test.

### 2.5. Enzyme-Linked Immunosorbent Assay

Antibody binding to PcCSP or PcTRAP in macaque plasma were measured by an IgG enzyme linked immunosorbent assay (ELISA) using previously described methods [18,19,20]. Briefly, Nunc Maxisorp Immuno ELISA plates coated with PcCSP or PcTRAP antigens diluted in PBS to a final concentration of 2 µg/mL and incubated at room temperature (RT) overnight. Plates were washed 6 times with PBS/0.05% Tween (PBS/T) and blocked with 300 µL with PierceTM protein-free (PBS) blocking buffer (Thermo Fisher Scientific, Waltham, MA, U.S.) for 2 h at RT. Macaque plasma was added and serially diluted 3-fold down in PBS/T with 50 µL per well as final volume and incubated for 2 h at RT. Following washing 6 times with PBS/T, bound antibodies were detected following a 1 h incubation with 50 µL of alkaline phosphatase-conjugated antibodies specific for monkey IgG (Sigma Aldrich, SLM, U.S.). Following additional 6 washes with PBS/T, development was achieved using 100 µL of 4-nitrophenylphosphate diluted in diethanolamine buffer and the absorbance values at OD405 were measured and c using a CLARIOstar instrument (BMG Labtech, Aylesbury, GB). Log reciprocal antibody titers were defined by an absorbance value three standard deviations greater than the average OD405 of the control. The antibody titers were analyzed by unpaired *t*-test to determine the *p*-values. 

### 2.6. Immunogenicity Responses and Challenge in Rhesus Macaque 

Before the start of the study, ethical approval was obtained from the animal ethics committee (Dierexperimentencommissie (DEC)) of the Biomedical Primate Research Centre (BPRC). The study protocol was registered under the DEC accession no. 745. All housing and animal care procedures took place at the BPRC in Rijswijk, the Netherlands, and were in compliance with European directive 2010/63/EU, as well as the Standard for Humane Care and Use of Laboratory Animals by Foreign Institutions provided by the Department of Health and Human Services of the United States National Institutes of Health (NIH, identification number A5539-01). The BPRC is accredited by the American Association for Accreditation of Laboratory Animal Care. Ten healthy purpose-bred rhesus macaques (4 female, 6 male) (*Macaca mulatta*) were stratified into two groups of five after which treatment was randomly assigned to each group. Stratification was based on sex, age, and body weight. All animal handling and biosampling was performed under ketamine sedation (10 mg kg^−1^, by intramuscular injection). At the end of the infection animals were euthanized by intravenous injection of pentobarbital (200 mg kg^−1^) under ketamine sedation. Vaccines were administered i.m. on days 0 and 56 (ChAdOx1 at dose of 1 × 10^10^ IU, MVA at dose of 1 × 10^8^ PFU respectively). Challenge was performed on day 70 by 10,000 *P. cynomolgi* sporozoites M strain administered i.v. The challenge data was analyzed and p-values were determined by the Kaplan–Meier method.

## 3. Results

### 3.1. Transgene Design in ChAdOx1 and MVA Vaccines Expressing TRAP and the CSP 

**Figure 1:**
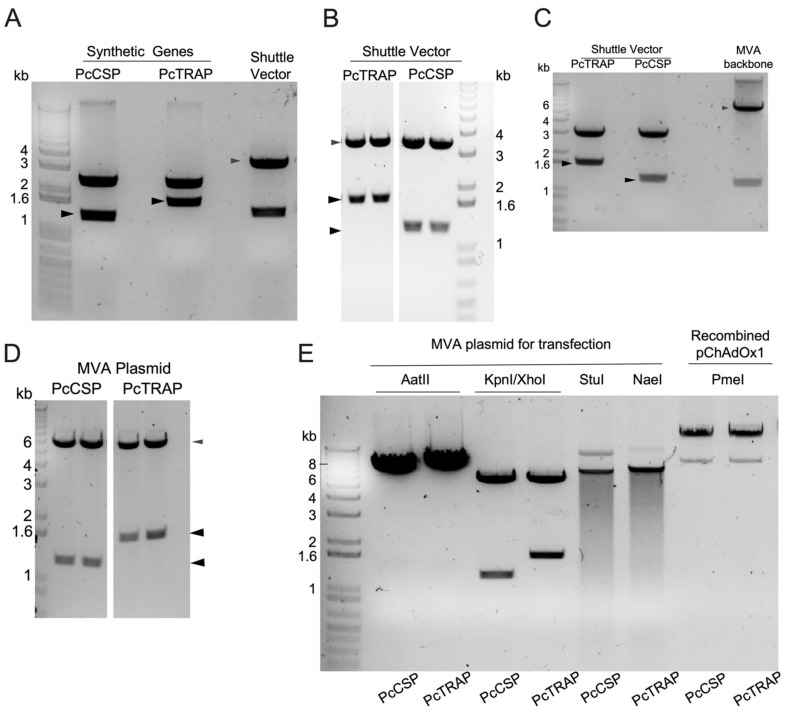
Design, cloning and production of ChAdOx1 and MVA viral vectored vaccines expressing *P. cynomolgi* (Pc) circumsporozoite protein (PcCSP) and Thrombospondin Related Adhesive Protein (PcTRAP). (**A**–**E**). (**A**) Agarose gel showing restriction fragment length polymorphism (RFLP) of the initial plasmids containing the synthetic genes of PcCSP and PcTRAP genes (**B**) Agarose gel showing PcCSP and PcTRAP bands upon enzyme restriction of from Shuttle Vectors and final virus plasmids. (**C**–**E**) Each cassette was cloned into a Shuttle vector and subsequently into both, adenoviral ChAdOx1 and Modified Vaccinia Ankara (MVA) vectors.

We constructed both, chimpanzee adenovirus (ChAdOx1) and MVA vaccines expressing the *Plasmodium cynomolgi* Circumsporozoite Protein (PcCSP) and Thrombospondin Related Adhesive Protein (PcTRAP). To this end, the annotated CSP sequences from *P. cynomolgi* strain B (Uniprot P08676; GeneBank AAA29539.1) and TRAP from *P. cynomolgi* (Uniprot 044019; GeneBank CAA73140.1) were used for the gene synthesis (Figure 1A). Upon enzymatic restriction of the initial PcCSP cassette, a band of 1,125 bp was visualized in a 1% agarose gel, whilst the PcCSP cassette yielded band corresponding to a size of 1,521 bp (Figure 1A). Each cassette was transferred to a Shuttle vector plasmid (pMONO) (Figure 1B) for subsequent cloning into an adenoviral (ChAdOx1) or to entry plasmid MVA which was co-transfected to produce and a Modified Vaccinia Ankara (MVA) plasmid vectors (Figure 1B–E). This resulted in the production of four viral vectored vaccines: ChAdOx1 PcCSP, ChAdOx1 PcTRAP, MVA PcCSP, and MVA PcTRAP. 

### 3.2. Design and Production of PcTRAP and the PcCSP Proteins

**Figure 2:**
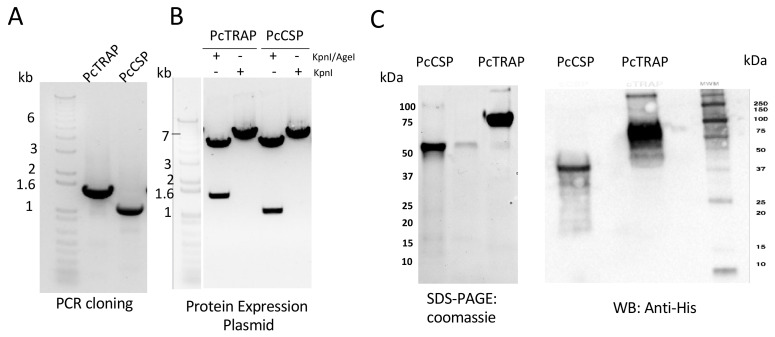
Design and cloning of PcCSP and PcTRAP transgenes for protein expression. (**A**) Agarose (1%) gel showing the PcCSP and PcTRAP PCR products following the restriction with AgeI and KpnI enzymes. (**B**) Agarose (1%) gel showing a single or double digestion with AgeI and KpnI to confirm the insertion of the PcCSP and PcTRAP into the PhLSec plasmid. (**C**) SDS-PAGE coomassie-stained gel (left) and Western blot (WB) with an anti-His monoclonal antibody (right) for detection of PcCSP and PcTRAP proteins upon transfection of HEK293 cells with PhLSec plasmids expressing either transgene.

We designed *PcCSP* and *PcTRAP* plasmid constructs for expression, production and purification of soluble PcTRAP and PcCSP proteins in mammalian system, to be used for the analysis of antibody responses. To this end, the *PcCSP* and *PcTRAP* genes were cloned into the pHLsec expression vectors to produce PcTRAP and PcCSP proteins with a C-terminal His tag (Figure 2A–B). These constructs were expressed in HEK293T cells and the secreted proteins were purified by nickel column chromatography. The purified proteins were analyzed by SDS-PAGE gel with coomassie staining and Western blot (WB) using an anti-His tag antibody (Figure 2C). Specific bands of 50 kDa and ~75 kDa were visualized, corresponding to PcCSP and PcTRAP proteins in WB respectively. 

### 3.3. Pre-Clinical Immune Responses after Vaccination in Mice

**Figure 3:**
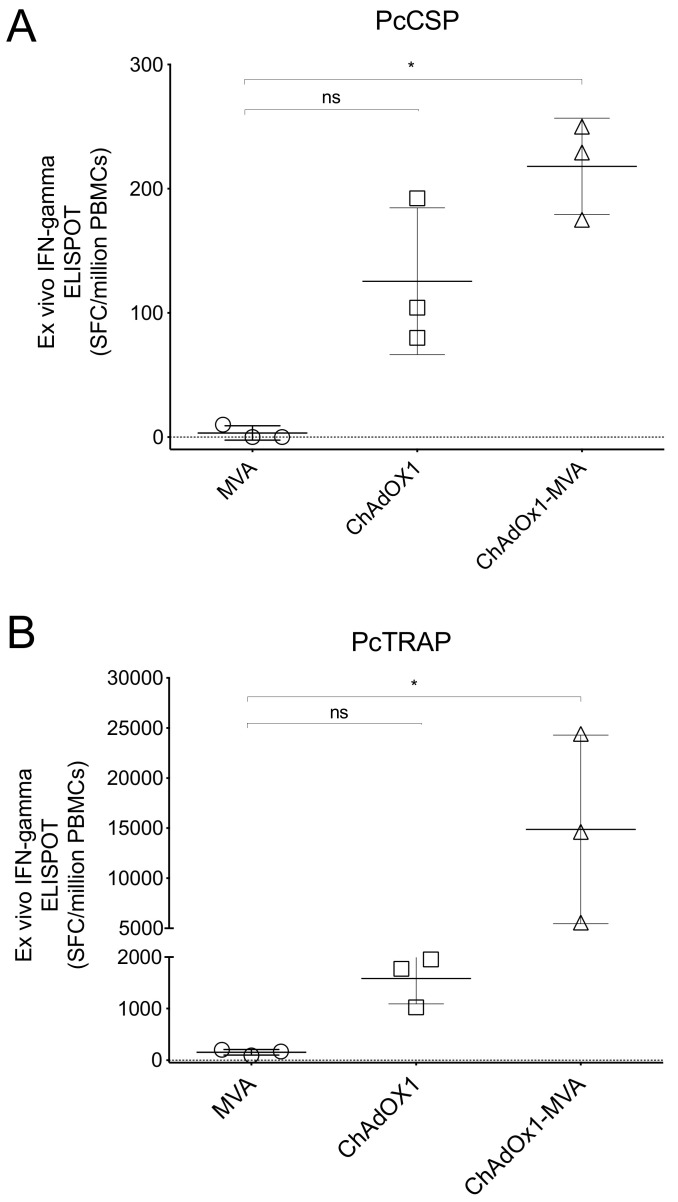
Cellular immune responses elicited by ChAdOx1 and MVA vaccines. C57Bl/6 mice (*n* = 3 per group) were injected with either ChAdOx1 PcCSP or ChAdOx1 PcTRAP at a concentration of 1 × 10^8^ IU/mouse, and MVA PcCSP and MVA PcTRAP at concentrations of 1 × 10^6^ PFU/mouse. A heterologous Prime-Boost using ChAdOx1-MVA vaccination regimen was also tested. Peripheral blood mononuclear cells (PBMCs) were cultured with peptide pools spanning the PcCSP and PcTRAP proteins and responses were assessed using an ex vivo IFN-gamma ELISpot. Values represent the spot-forming cells (SFC) per million PBMCs resulting from stimulation with 20-mer overlapping by 10 peptides spanning the whole PcCSP (**A**) and PcTRAP (**B**) structural proteins (10 µg/mL). Line shapes represent mice vaccinated with each vaccine. *p*-values were determined by Kruskal–Wallis test and Dunn’s multiple comparisons test. *p* > 0.05 (ns), *p* < 0.05 (*).

An immune potency assay was performed in mice in order to assess immunogenicity of our new viral vectored vaccines prior to the rhesus trial. C57Bl/6 mice (*n* = 3) were immunized with either ChAdOx1 PcCSP or ChAdOx1 PcTRAP using a standard dose of 1 × 10^8^ IU [21]. Similarly, MVA PcCSP and MVA PcTRAP were assessed for immunogenicity in the same animal model, using standard doses of 1 × 10^6^ PFU/mouse [22]. Mock control mice received a boost with MVA with twice the concentration (2 × 10^6^ PFU/mouse) to match the amount of MVA in the other groups. 2 weeks after immunization, T cell responses were quantified by an ex vivo IFN-gamma ELISpot, by stimulating Peripheral Blood Mononuclear Cells (PBMCs) with a specific pool of overlapping peptides spanning the PcCSP or PcTRAP proteins (Figure 3). C57Bl/6 mice immunized with MVA PcCSP and PcTRAP induced low T-cell responses, as described in previous reports [23]. C57Bl/6 mice receiving the ChAdOx1 PcTRAP vaccine showed good T cell frequencies of ~1500 spot-forming cells (SFC)/10^6^ PBMCs (Figure 3B) while ChAdOx1 PcCSP vaccine induced only modest mean T cell frequencies of ~130 spot-forming cells (SFC)/10^6^ PBMCs (Figure 3A). The T cell responses increased significantly to reach mean T cell frequencies of ~15,000 SFC/10^6^ PBMCs by prime-boost vaccination (ChAdOx1-MVA) of PcTRAP, whereas mice receiving a prime-boost vaccination of ChAdOx1-MVA PcCSP, reached a mean of ~200 SFC/10^6^. Whilst we confirmed that all viral vectors were immunogenic, we observed a modest immunogenicity by PcCSP when compared to that PcTRAP vectored vaccines were more immunogenic than the PcCSP counterpart. 

### 3.4. Humoral and Cellular Immune Responses after Vaccination in Rhesus Macaque Prior to Challenge

**Figure 4:**
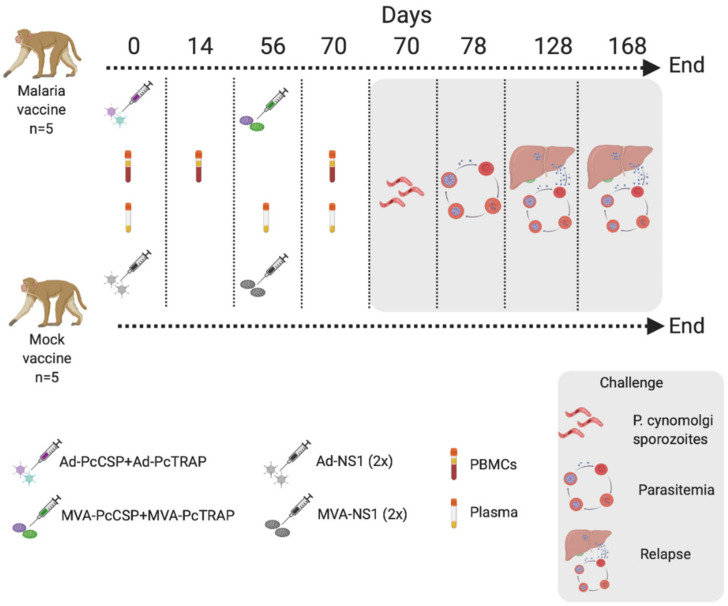
Description of Nonhuman Primates (NHP) challenge experiment. Created with BioRender.com.

A macaque trial was designed to run for 168 days (Figure 4), during which nonhuman primates (NHPs) were vaccinated on day 0 with a combination of ChAdOx1-PcCSP + ChAdOx1-PcTRAP (Malaria group, *n* = 5) or ChAdOx1-NS1 (Mock control group receiving a 2x of a dengue NS1 vaccine, *n* = 5) (Figure 4). NHPs received a boost with MVA-PcCSP + MVA-PcTRAP or an MVA-NS1. Blood samples were taken on days 0, 14, and 70 (PBMCs) or 0, 56 and 70 (Plasma). All NHPs were challenged with 10,000 *P. cynomolgi* sporozoites delivered intravenously and followed up to assess parasitemia from day 78, treated with chloroquine to eliminate blood stage parasitemia and followed up to detect two relapses up until day 168 when the experiment was terminated. 

**Figure 5:**
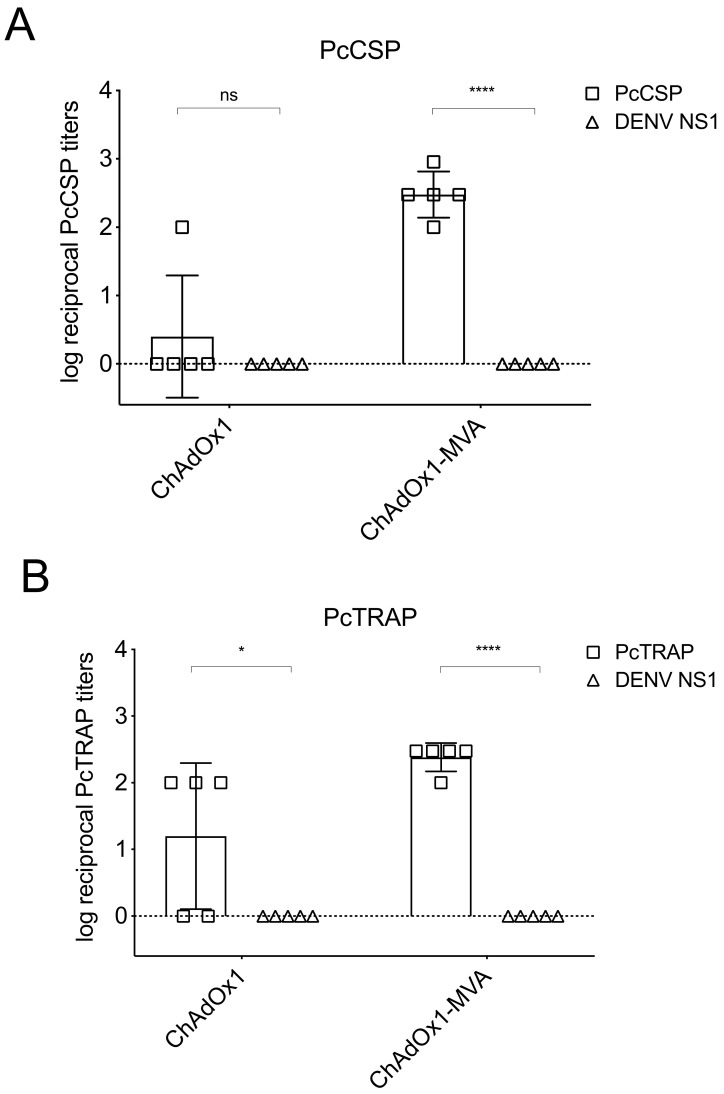
Humoral immune responses elicited by ChAdOx1/MVA vaccines in rhesus macaques. A group of rhesus macaques (*n* = 5) were co-immunized with ChAdOx1 PcCSP and ChAdOx1 PcTRAP at 1 × 10^10^ iu and the control group (*n* = 5) were immunized with unrelated ChAdOx1 DENV NS1 vaccine. Eight weeks after prime vaccination, the animals were boosted with MVA encoding the same antigens. Plasma samples were collected at 8 weeks after prime immunization and 2 weeks after P-B immunization. Antibody responses elicited by vaccines were quantified by ELISA in plates coated with a PcCSP (**A**) or PcTRAP (**B**) proteins. *p*-values were determined by unpaired t-test. *p* > 0.05 (ns), *p* < 0.05 (*), *p* < 0.01, *p* < 0.001, *p* < 0.0001 (****).

Prior to the rhesus macaque challenge, we assessed vaccine-elicited immunogenicity after the prime or the prime-boost (Figure 5). At 8 weeks post-prime immunization, only 1 out of 5 animals seroconverted for PcCSP (Figure 5A) while the anti-PcTRAP antibodies were detected in 3 out of 5 animals at the log reciprocal antibody titer values of 2 (Figure 5B). Two weeks after the boost immunization, the log reciprocal antibody titer values for both anti-cCSP and anti-cTRAP increased significantly to ~2.47 and ~2.38 respectively (Figure 5A,B). Anti-cCSP and anti-cTRAP antibodies, in plasma from the control group, were negative in all timepoints.

**Figure 6:**
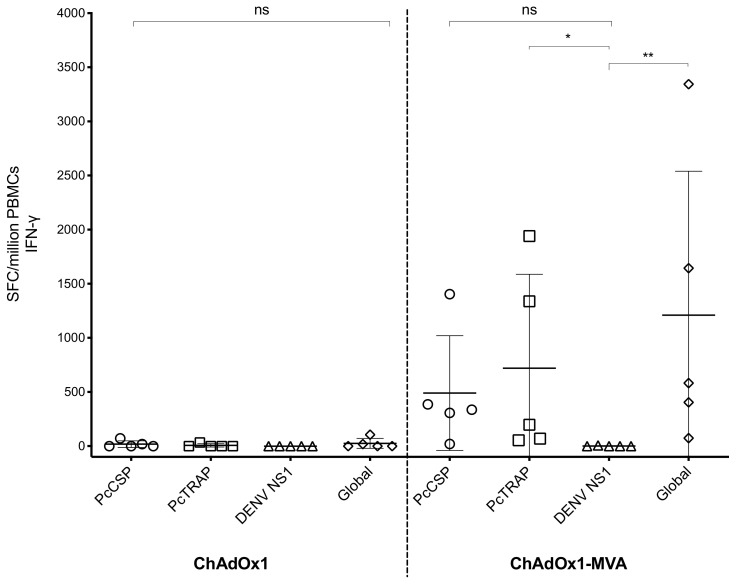
T cell immune responses elicited by ChAdOx1/MVA vaccines in rhesus macaques by ELISpot. PBMCs were cultured with peptide pools spanning the PcCSP and PcTRAP proteins and responses following prime and prime-boost immunizations were assessed using an ex vivo IFN-gamma ELISpot. Values represent the spot-forming cells (SFC) per million PBMCs resulting from stimulation with 20-mer overlapping by 10 peptides spanning the whole PcCSP and PcTRAP structural proteins (10 µg/mL). Individual data are shown as symbols and mean + SD are represented as the horizontal bars. PBMC samples were collected at 2 weeks after prime immunization and 2 weeks after P-B immunization. *p*-values were determined by Kruskal–Wallis test and Dunn’s multiple comparisons test. *p* > 0.05 (ns), *p* < 0.05 (*), *p* < 0.01 (**).

Antigen-specific cellular T responses were quantified using an ex vivo IFN-gamma ELISpot assay from PBMCs (Figure 6). Results showed that only small frequencies of T cell responses were induced after priming with ChAdOx1-PcCSP+PcTRAP. T cell responses increased to reach mean T cell frequencies of ~490 and ~720 SFC/10^6^ PBMCs for PcCSP and PcTRAP, respectively with a mean overall response of ~1210 SFC/10^6^ PBMCs when adding T cell responses for both antigens. We concluded that good humoral and cellular responses were induced after prime-boost vaccination of rhesus macaques prior to sporozoite challenge. These results conclude that good humoral and cellular responses were induced in all prime-boost vaccinated group of rhesus macaques (*n* = 5) prior to sporozoite challenge.

### 3.5. Challenge of NHPs with P. cynomolgi sporozoites

**Figure 7:**
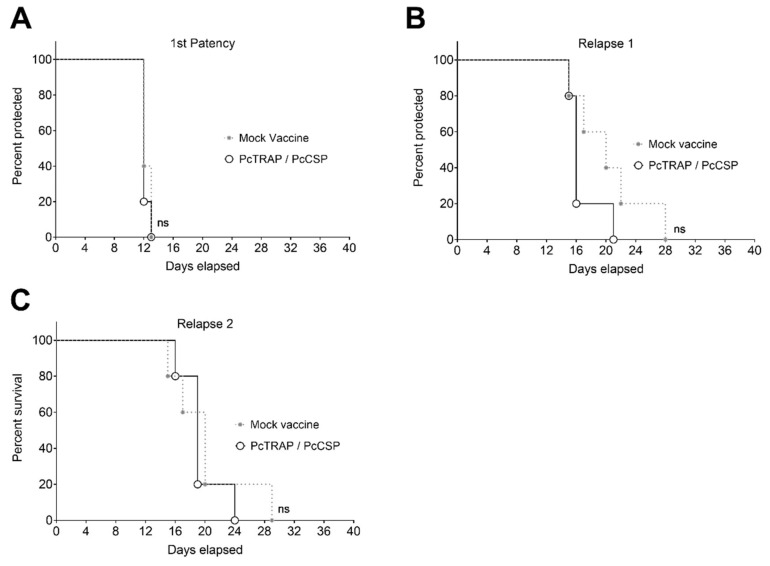
Macaques challenge with 10000 *P. cynomolgi* sporozoites. Macaques were followed to assess time and number of relapses; (**A**) 1^st^ patency, (**B**) First relapse and (**C**) Second relapse. Survival curves are represented as the time when the relapses occur. *p*-values were determined by the Kaplan–Meier method. *p* > 0.05 (ns).

Macaques were challenged with 10,000 *P. cynomolgi* sporozoites following an MVA boost (Figure 4). When parasitemia became patent (thin smear positive), macaques were treated with chloroquine to eliminate only the blood-stage parasites without affecting hypnozoites. Macaques were then followed-up for a maximum of 24 weeks to assess time and number of relapses and determine whether a difference between control and vaccinated animals could be detected. There were no significant differences between mock-control and vaccinated animals, neither for the time taken for 1st patency or the time or number of relapses, which suggest that the vaccination with ChAdOx1 or MVA viral vectors expressing PcTRAP or PcCSP do not protect against pre-erythrocytic malaria or relapses (Figure 7A–C).

## 4. Discussion

*P. vivax* presents a more challenging landscape than *P. falciparum* for vaccine development, due to the need to prevent relapses caused by hypnozoites in the liver. Vaccine clinical trials for *P. falciparum* have moved decisively forward, and leading pre-erythrocytic *P. falciparum* candidates (R21, RTS, S, and Ad-M ME.TRAP) based on CSP and TRAP proteins alone or in combination have been tested in a control human malaria infection with encouraging results showing that such vaccines offer partial protection against malaria sporozoite challenge on their own [24]. Results indicate that protective immunity against malaria may require the stimulation of strong humoral and cellular responses against more than one antigen [25]. Our previous studies have shown that tailoring a combination of these two pre-erythrocytic antigens (CSP and TRAP) could enhance protection to mouse sporozoite challenge in mouse malaria models using *P. berghei* and *P. vivax* antigens [13,22]. In this study, we constructed two *P. cynomolgi* vaccines encoding the PcCSP and PcTRAP antigens based on viral-vector platforms (ChAdOx1 and MVA vectors) to address the question whether these pre-erythrocytic antigens expressed in viral vectored vaccines can prevent relapse in non-human primates (NHP). To assess the potency of our vaccines prior to the NHP immunizations and challenge, the immunogenicity of our viral-vectored vaccines was first assessed in mice by measuring cellular response in C57Bl/6 mice which suggested that MVA PcCSP and PcTRAP vaccines immunized as prime induced low T-cell responses as described previously [23] while the ChAdOx1 vaccine counterpart demonstrated significantly higher T cell responses as previously shown by other ChAdOx1 vaccines encoding various different antigens [18]. Interestingly, PcTRAP vaccines stimulated higher T cell frequencies than PcCSP regardless of whether it was given as a prime (ChAdOx1) or prime-boost (ChAdOx1-MVA) regimen, which is consistent to the observations made previously in *P. vivax* CSP and TRAP publications where no T-cell epitopes have been identified for CSP as opposed to multiple T-cell epitopes for TRAP [12,21,26]. Our results show that despite induction of humoral and cellular responses in all vaccinated group of NHPs immunized with ChAdOx1–MVA (PcCSP/PcTRAP), there was no effect in protection against *P. cynomolgi* sporozoite infection or relapse, as shown by the development of the parasite blood-stage in both scenarios. The challenge was carried out using a high challenge dose of 10,000 *P. cynomolgi* sporozoites in order to make it a very stringent test and this may account for the lack of protection in the challenge model. It has been shown by later studies using the same model that the challenge can be performed reproducibly with 500–1000 sporozoites which is much less than the number of sporozoites used in this study.

Recent studies published by the start of this work have shown a reduction of anti-CSP antibodies as well as the reduction in efficacy when both, CSP and TRAP were delivered by viral-vectored vaccines, contrary to an adenovirus prime and protein–CSP boost regimen led to increased anti-CSP antibody titers as well as optimal protection in mouse model [13]. It is possible that the heterologous ChAdOx1 and MVA prime-boost strategy (ChAdOx1–MVA used in this study led sub-optimal induction of PcCSP and PcTRAP specific antibodies and antigen specific T-cell responses that failed to provide any protection in the NHP challenge model. Therefore, alternative vaccination strategies such as the adenovirus prime and protein PcCSP boost regimen could be tested to determine if such strategy could increase the anti-PcCSP titers and efficacy in the future. We have recently shown that a VLP based on Hep B Surface antigen (Rv21) provided 100% sterile protection against *P. berghei*/Pv CSP transgenic sporozoite challenge in rodent malaria models [21] and this scenario indicates that it would be interesting to generate a *P. cynomolgi*–specific VLP to assess if relapse could be prevented. Another recent study tailoring a combination of *P. vivax* CSP+TRAP vaccine has shown that a combination of VLP (PvCSP)+ viral vectors (PvTRAP) was superior in efficacy against infection to that of viral vectors expressing both antigens [22] and thus, such combination could also to be tested in the future. Furthermore, it was recently shown that elimination of the N-terminus from CSP in a VLP can enhance protecting efficacy in mouse challenge model (Atcheson et al. Vaccine. In press). Therefore, it would be interesting to eliminate the N-terminus from PcCSP to assess if this enhances protective efficacy. These results were not available at the time when our vaccines were tested in NHP challenge model described in this work, but they will be invaluable for generation of new vaccine candidates that could be tested by a future NHP trial. Interestingly, major advances to support *P. falciparum* and *P. vivax* vaccine development have been the availability of transgenic parasites, in which a target protein vaccine candidate of either parasite can be inserted into *P. berghei*, to allow testing efficacy in mouse models. Our results highlight the importance of the generation of *P. berghei* transgenic parasites expressing *P. cynomolgi* transgenes to assess efficacy of new vaccines before taking candidates to NHP trials, in an attempt to select the best candidates. 

## 5. Conclusions

This study reports the development of viral vectored vaccines consisting of ChAdOx1 and MVA platforms expressing *P. cynomolgi* PcCSP and PcTRAP vaccine candidates, as well as the expression of PcCSP and PcTRAP proteins in mammalian cell culture. We have demonstrated that despite induction of humoral and cellular responses against both PcCSP and PcTRAP in NHP prior to the sporozoite challenge, neither infection, nor relapse were prevented. This prompts towards further research in the generation of novel vaccine platforms, such as the leading VLPs based on Hepatitis B Surface antigen like Rv21 for *P. vivax* and its combination with additional antigens to explore relapse prevention.
